# The Impact of Precisely Controlled Pre-Freeze Cooling Rates on Post-Thaw Stallion Sperm

**DOI:** 10.3390/ani16010021

**Published:** 2025-12-21

**Authors:** Aviv Bitton, Amos Frishling, Dorit Kalo, Zvi Roth, Amir Arav

**Affiliations:** 1 Koret School of Veterinary Medicine, The Hebrew University of Jerusalem, Rehovot 7610001, Israel; 2 Equine Reproduction Laboratory, Bnei Zion 6091000, Israel; 3 Department of Animal Sciences, Robert H. Smith Faculty of Agriculture, Food and Environment, The Hebrew University of Jerusalem, Rehovot 7610001, Israel; 4 Miller School of Medicine, University of Miami, Miami, FL 33136, USA

**Keywords:** stallion spermatozoa, cryopreservation, directional freezing, sperm motility, cold shock

## Abstract

Cryopreservation is a key tool in assisted reproduction, but it often compromises post-thaw sperm quality due to cryodamage. Optimizing the initial cooling phase, specifically from room temperature to 5 °C, is a critical determinant of successful outcomes. This study aimed to evaluate the impact of different pre-freeze cooling rates on stallion sperm quality using a novel, precision cooling device. Semen samples from five healthy stallions were divided into three groups and cooled at distinct rates: Slow (0.3 °C/min), Moderate (1 °C/min), and Fast (approximately 30 °C/min). Sperm motility parameters were assessed using a Computer-Assisted Sperm Analyzer (CASA) before freezing and after thawing. Additionally, sperm integrity and physiological parameters, including viability, acrosomal integrity, Reactive Oxygen Species (ROS) expression, and mitochondrial membrane potential, were assessed by flow cytometry post-thaw. The analysis of post-thaw kinematics revealed a significant interaction between the cooling rate and processing stage (post-cooling vs. post-thaw). The Fast-cooling protocol resulted in higher post-thaw total motility (51.8%) compared to the Slow protocol (45.01%). Crucially, no significant differences were detected among cooling rates for the critical parameter of progressive motility or curvilinear velocity (VCL). Circle motility had higher values in the Fast-cooling group compared to the Slow group. Cell viability demonstrated a tendency (*p* = 0.08), where the Slow cooling group exhibited higher mean values (65.59%) compared to the Fast group (61.67%). Comprehensive flow cytometry assessments of other cellular integrity markers, including acrosomal integrity, mitochondrial function (MMP), and ROS expression, were statistically equivalent across all cooling rates (*p* > 0.05). The results confirm that this fast pre-freeze cooling rate, integrated within the highly controlled environment of Directional Freezing technology, successfully preserved essential sperm function and structure. Critically, the demonstrated functional equivalence in progressive motility validates the Fast protocol as an efficacious strategy to increase the efficiency and adaptability of equine semen cryopreservation protocols for commercial utilization.

## Simple Summary

Freezing stallion semen is crucial for global breeding, but it is traditionally a slow process to avoid “cold shock” damage to the sperm. This study tested a new, precise cooling device to see if sperm could survive faster cooling rates. Surprisingly, we found that sperm cooled rapidly (in minutes rather than an hour) maintained excellent quality and fertility potential, comparable to the traditional slow method. This discovery is significant because it allows laboratories to integrate the initial cooling directly into the freezing machine. This creates a streamlined “one-stop” process that saves time and money, making high-quality artificial insemination more efficient for the equine industry.

## 1. Implications

Successful freezing of horse semen traditionally requires slow cooling protocols, making the process time-consuming for breeding operations. Our study used precision technology to show that semen maintains excellent quality even when cooled extremely quickly (25–30 °C/min). The success of this high-precision cooling stage validates the practicality of a streamlined, highly accelerated cryopreservation procedure, allowing the initial cooling to be integrated into the Directional Freezing technology. This technological advancement improves laboratory workflow and operational efficiency, reducing the overall cost and complexity of producing high-quality frozen stallion semen for the global equine industry.

## 2. Introduction

Cryopreserved semen is a cornerstone of the equine breeding industry, particularly revolutionizing the dissemination of elite sport horse genetics globally and enabling the long-term storage of valuable genetic material [1,2]. This technology enhances breeding programs by simplifying breeding management. However, the cryopreservation of stallion spermatozoa remains notoriously challenging. A prominent and frustrating characteristic of the field is the significant variation in post-thaw sperm survivability between individuals- the so-called “good” vs. “poor freezer” problem [3,4]. This inherent variability has limited the widespread clinical adoption of the technology, remaining the primary obstacle to overcome. Some estimates suggest only 25% of stallions achieve acceptable pregnancy rates with frozen-thawed semen, while up to 30% perform very poorly [5,6]. This necessitates ongoing research to develop standardized protocols that can overcome this individual variability [7].

The cryopreservation process exposes sperm cells to a harsh, hyper-osmotic environment, initiating a cascade of damaging events collectively known as cryodamage. Cryodamage occurs across two distinct thermal phases: “cooling injury” (or cold shock) above 0 °C and physical/osmotic damage during freezing below 0 °C [8,9]. Cold shock is defined as the irreversible damage caused by exposure to low temperatures without freezing [10,11]; this phenomenon was linked to a lipid phase transition in the sperm plasma membrane. As the temperature drops, lipids undergo a phase transition, shifting from a fluid-crystalline state to a more rigid, gel-like state [12]. Specifically, cooling rates faster than approximately 0.3 °C/min between 20 °C and 8 °C have been shown to induce irreversible cold shock in stallions, leading to severe consequences [13,14,15]. Subsequently, during the freezing stage, the formation of both intracellular and extracellular ice crystals causes direct physical damage to the cell and organelles [16]. Beyond physical disruption, cryopreservation also induces significant molecular and epigenetic harm, such as increased DNA methylation, which may contribute to lower pregnancy rates independent of standard metrics [17,18].

Maximizing post-thaw cell survival requires careful optimization of the cryoprotective agent (CPA) composition, the extender formulation, the cooling rate, and the warming (thawing) rate [4,19]. The relationship between these physical parameters is crucial, as cell survival is generally considered a biphasic function of the cooling rate [8,12]: Slow cooling causes excessive dehydration (known as “solution effect”), while Fast cooling leads to intracellular ice crystal formation [20,21]. Glycerol is the most widely used CPA, and while it is essential for reducing the freezing point, it can become toxic at high concentrations or under prolonged exposure times [22]. While freezing protocols (from 5 °C downwards) are well-established [14], the initial cooling phase (room temperature to 5 °C) remains a critical but under-optimized aspect of stallion semen cryopreservation. Established protocols have traditionally advocated very slow cooling rates during this phase [13,23]. A precisely controlled rate is essential not just for avoiding cold shock, but also for proper water transport dynamics, allowing the CPA (e.g., glycerol) sufficient time for cell equilibration, thereby minimizing osmotic damage [24,25]. The conceptual shift from passive “damage management” to a proactive approach that precisely controls the physical cause of injury has been robustly validated during the freezing phase itself. Specifically, the development of directional freezing technology, utilizing the Multi-Thermal Gradient (MTG) device, has demonstrated superior survival by precisely controlling ice crystal morphology and growth [26,27,28]. Applying this proven principle of highly controlled physical manipulation to the initial cooling phase is a logical and promising next step. However, conventional pre-freeze methods lack the precision to apply this principle effectively.

Utilizing a novel, custom-built precision cooling device with accurate and repeatable thermal control, this study systematically investigated the effects of three distinct pre-freeze cooling rates—Slow (0.3 °C/min), Moderate (1 °C/min), and Fast (approx. 25–30 °C/min)—on post-thaw stallion spermatozoa quality. Post-thaw sperm quality was comprehensively assessed, including motility parameters and key biochemical integrity markers (viability, mitochondrial membrane potential, acrosomal status, and intracellular ROS expression) [29]. The inclusion of rates traditionally considered detrimental [13,30] allows for a re-evaluation of these limits under the precisely controlled conditions afforded by our technology. We hypothesized that a precisely controlled Moderate cooling rate (1 °C/min) could provide a time-efficient protocol that preserves sperm quality comparable to the conventional Slow rate (0.3 °C/min), offering a significant practical advantage.

## 3. Materials and Methods

### 3.1. Experimental Design

The experimental unit was defined as the individual semen ejaculate. Each ejaculate was split into three aliquots, one for each cooling protocol, resulting in a total of 45 observations derived from 15 independent ejaculates (*N* = 15). This sample size was determined to be adequate based on an a priori power analysis to ensure sufficient statistical power to test the primary hypothesis. The core methodology involved splitting each ejaculate into three aliquots, which were then randomly assigned to be processed using one of three defined pre-freeze cooling rate protocols (Slow, Moderate, or Fast), thereby utilizing each ejaculate as an internal control (block).

### 3.2. Animals

Semen was collected from five clinically healthy, sexually mature Quarter Horse stallions, aged 8 to 14 years, located on several commercial farms throughout Israel. Stallions were selected based on their active participation in a commercial artificial insemination program and high initial fresh semen quality (total motility > 50%). No prior information regarding their specific freezability classification was used as a selection criterion. A total of 15 ejaculates were obtained for the study (three ejaculates per stallion). All collections were performed by licensed veterinarians as part of the stallions’ routine commercial breeding activities, with the informed consent of the owners. The procedures adhered to standard veterinary practices and ethical guidelines.

### 3.3. Semen Collection and Initial Evaluation

Semen was collected using a Missouri model artificial vagina (AV). Immediately after collection, the gel fraction was removed, the semen was filtered, and then immediately diluted at a 1:1 ratio with a commercial, milk-based transport extender pre-warmed to 37 °C, containing 5% glycerol (Inra 96, Ref. 016441, IMV-Technologies, L’Aigle, France). An initial assessment of sperm concentration and total motility of the diluted sample was performed using a Computer-Assisted Sperm Analyzer (CASA; AndroScope, Minitube, Tiefenbach, Germany).

### 3.4. Semen Processing

Following the initial evaluation, samples were transported to the laboratory in an insulated container, where all subsequent processing was conducted at room temperature (approx. 22 °C). For centrifugation, diluted semen was layered over a 2 mL density cushion (Maxifreeze, Ref. 019269, IMV-Technologies, L’Aigle, France) in a 50 mL conical tube and centrifuged at 800× *g* for 12 min. The resulting sperm pellet was isolated by discarding the supernatant and cushion medium and then resuspended in a commercial egg yolk-based freezing extender (Inra Freeze, Ref. 022110, IMV Technologies, L’Aigle, France) to a final concentration of approx. 200 × 10^6^ spermatozoa/mL. The resuspended semen was then divided into three equal aliquots. Prior to cooling, each aliquot was loaded into labeled 0.5 mL plastic straws and sealed with metal balls.

### 3.5. Cooling Protocols

Pre-freeze cooling was performed using a custom-built thermoelectric (Peltier) device (A.A.Cash Technology Ltd II, Tel Aviv, Israel). This unit features a conductive cooling block specifically designed for 0.5 mL straws, enabling precise, continuously monitored temperature control. For the Slow and Moderate cooling rates, the device was programmed to cool the straws from room temperature (approx. 22 °C) to 5 °C at a linear rate of 0.3 °C/min and 1 °C/min, respectively. For the Fast-cooling protocol, the cooling block was pre-cooled to 5 °C; straws were then placed directly into the pre-cooled block, resulting in a non-linear cooling curve that reached 5.5 °C in under 40 s (approx. average rate of 25–30 °C/min). The cooling rates were selected to investigate the effects of cold shock.

### 3.6. Cryopreservation and Thawing

Following the pre-freeze cooling to 5 °C, straws were cryopreserved in a programmable freezer (Multi-Thermal Gradient, MTG-16, IMT Ltd., Ness Ziona, Israel) as previously described [28,31]. The device operates on the principle of directional freezing, where straws are moved along a thermal gradient created by a series of blocks. In this study, the device was configured with three blocks set at 5 °C, −50 °C, and −70 °C, and programmed with a freezing velocity of 1.5 mm/s, a seeding distance of 10 mm, and a seeding time of 20 s. Following completion of the freezing program, straws were plunged into liquid nitrogen (−196 °C) for storage. For post-thaw motility assessment and biochemical analysis, one straw from each experimental group was thawed in a 37 °C water bath for 30 s.

### 3.7. Sperm Motility Assessment

The motility analysis was conducted using a Computer-Assisted Sperm Analyzer (CASA; Androscope, Minitube). Sperm motility was assessed at two time-points: immediately following the completion of each cooling protocol (pre-freeze analysis) and after thawing (post-thaw analysis). Prior to assessment, samples were equilibrated for 10 min at 37 °C before an aliquot was loaded into a 20 µm depth analysis chamber slide for immediate evaluation. All analyses were conducted under standardized 37 °C conditions, using a 10x negative phase-contrast objective with image acquisition at 100 frames per second. For each analysis, a minimum of 3000 spermatozoa were evaluated across at least four different fields. The following parameters were recorded: total motility (TM), progressive motility (PM), fast motility, slow motility, circle motility, local motility, and curvilinear velocity (VCL).

### 3.8. Physiological Assays

All flow cytometry tests were performed using a Guava easyCyte™ microcapillary flow cytometer with CytoSoft software v 3.0 (Guava Technologies Inc., distributed by IMV Technologies). The following sperm parameters were assessed with lyophilized fluorochrome-containing kits (EasyKit; IMV Technologies, L’Aigle, France) as previously described [32,33]. For each analysis, data acquisition was stopped after 5000 sperm-specific events were recorded.

#### 3.8.1. Sperm Viability (Membrane Integrity)

Sperm viability was assessed using EasyKit 1 Viability and Concentration (ref. 024708; IMV Technologies, L’Aigle, France), which combines two fluorescent dyes to differentiate between sperm with intact (viable) and damaged (non-viable) plasma membranes. For analysis, 5 µL of the sperm sample was added to a well containing 200 µL of PBS and incubated for 10 min at 37 °C in the dark before acquisition. The results are expressed as the percentage of viable spermatozoa.

#### 3.8.2. Mitochondrial Membrane Potential

Mitochondrial membrane potential was assessed using EasyKit 2 (ref. 024864; IMV Technologies, L’Aigle, France). This kit contains a lipophilic dye that distinguishes between spermatozoa with active (high-potential, polarized) and inactive (low-potential, depolarized) mitochondria. For analysis, 5 µL of the sperm sample was added to a well containing 200 µL of PBS and incubated for 30 min at 37 °C in the dark before acquisition. The results are expressed as the ratio between the percentages of spermatozoa expressing polarized and depolarized mitochondrial membranes.

#### 3.8.3. Oxidation Status

The intracellular ROS expression was evaluated using EasyKit 3 (ref. 025157; IMV Technologies, L’Aigle, France). For the analysis, 5 µL of the sperm sample was added to a well containing 200 µL of prewarmed PBS (37 °C) and incubated for 20 min at 37 °C in darkness. Subsequently, 2 µL of 39 mM hydrogen peroxide was added to each well, followed by an additional incubation for 40 min at 37 °C. Samples were then washed with 600 µL of prewarmed PBS and centrifuged for 5 min at 300× *g*. The resulting pellet was resuspended in 200 µL of PBS, transferred back into a 96-well plate, and loaded into the flow cytometer for acquisition. The results were expressed as the percentage of viable spermatozoa with high or low ROS expression (i.e., ROS+-spermatozoa).

#### 3.8.4. Acrosome Integrity

The status of the acrosomal membrane was evaluated using EasyKit 5 (ref. 025293; IMV Technologies, L’Aigle, France). This kit uses a green probe to label spermatozoa with disrupted acrosomes, in combination with a viability dye. For analysis, 5 µL of the sperm sample was added to a well containing 200 µL of PBS and incubated for 45 min at 37 °C in the dark before acquisition. The results were expressed as the spermatozoa percentage with an intact or damaged acrosomal membrane.

### 3.9. Statistical Analysis

Statistical analysis was performed using R Studio (Version 4.3.1, RStudio Inc., Boston, MA, USA). Due to the non-normal distribution of the data, an Aligned Rank Transform (ART) ANOVA was conducted using the ARTool package. The model evaluated the main effects of cooling rate (Fast, Moderate, Slow), processing stage (Post-Cooling, Post-Thaw), and their interaction. To account for the repeated measures design, each individual ejaculate was included as a random effect in the model. This statistical model was applied to all sperm motility and flow cytometry parameters. When significant effects (*p* < 0.05) were detected, post hoc pairwise comparisons were performed using the art.con function with Tukey’s method for *p*-value adjustment.

## 4. Results

### 4.1. Initial Semen Characteristics

Fifteen raw ejaculates were collected and processed across experimental procedures. The mean initial sperm concentration was 151.27 ± 11.01 × 10^6^ spermatozoa/mL, with a median of 171.02 × 10^6^ spermatozoa/mL. Total motility and progressive motility prior to processing averaged 87.63 ± 1.72% and 77.82 ± 3.06%, respectively, indicating high baseline semen quality. Curvilinear velocity (VCL) averaged 142.36 ± 8.04 μm/s (Table 1).

### 4.2. Motility and Kinematic Parameters

The effects of the different cooling protocols on sperm motility and kinematic parameters, as assessed by CASA, are summarized in Table 2. Consistent with expectations regarding the deleterious effects of cryopreservation, a significant reduction was observed across all measured parameters following the freeze–thaw process (*p* < 0.001 for all samples). The subsequent analysis focused exclusively on the differential effects of the cooling rate protocols on post-thaw quality.

#### 4.2.1. Total Motility

**Figure 1:**
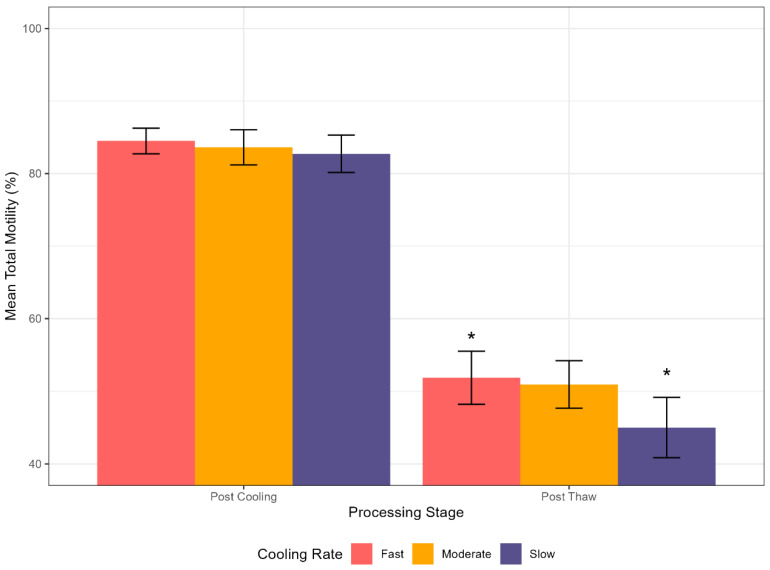
Interaction effect of pre-freeze cooling rate and processing stage on the total motility of stallion spermatozoa. The figure illustrates the mean total motility (%) measured after the initial cooling (Post Cooling) and after thawing (Post Thaw) for three different cooling protocols. Data are presented as mean ± SEM. The cooling rates were defined as: Fast (25–30 °C/min), Moderate (1 °C/min), and Slow (0.3 °C/min). An asterisk (*) denotes a statistically significant difference (*p* < 0.05) between the fast and slow cooling rates at the post-thaw stage.

A statistically significant interaction was identified between the processing stage (Post-Cooling vs. Post-Thaw) and the cooling rate for total motility (*p* < 0.05). While no significant differences in motility were observed immediately following the cooling stage (prior to freezing), the post-thaw assessment yielded a counter-intuitive outcome: the fast cooling rate resulted in significantly higher total motility (51.87%) compared to the Slow cooling rate (45.01%) (*p* < 0.05; Figure 1).

#### 4.2.2. Progressive Motility and Kinematics

**Figure 2:**
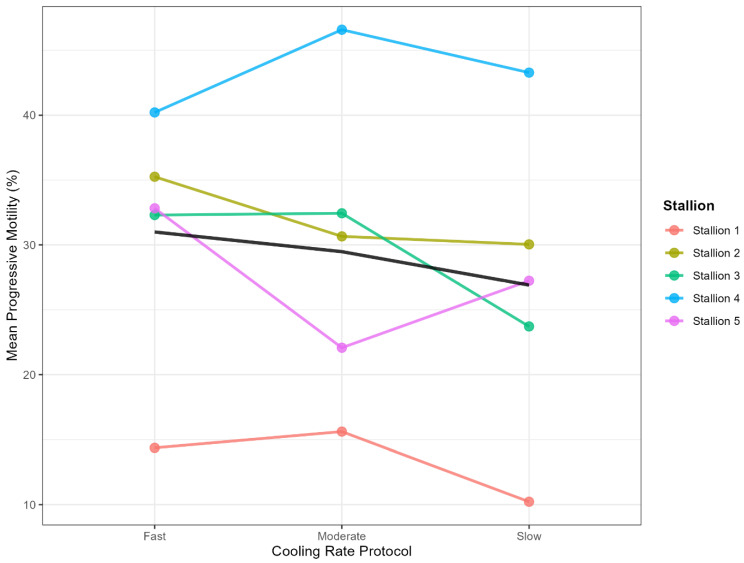
Individual variation in post-thaw progressive motility in response to different pre-freeze cooling rates. Each colored line represents the mean response of an individual stallion, while the black line indicates the overall mean response across all samples. The cooling rates were defined as: Fast (25–30 °C/min), Moderate (1 °C/min), and Slow (0.3 °C/min).

For the majority of other motility and kinematic parameters, including progressive motility, fast motility, and VCL (Velocity Curvilinear), no statistically significant differences were detected in the main effect of the cooling rate or its interaction with the processing stage (*p* > 0.05 for all comparisons). However, a noteworthy trend was observed for circle motility, which approached the threshold for statistical significance (*p* = 0.06). This kinematic pattern, which is sometimes associated with cold shock or altered membrane integrity, showed numerically higher post-thaw values in the fast cooling group (1.67%) compared to the Slow cooling group (1.18%), suggesting a potential, albeit subtle, differential effect of the fastest protocol on specific movement characteristics. In addition to the main effect, noticeable variation between individual ejaculates was also observed (Figure 2).

### 4.3. Sperm Physiological Markers

To ensure a comprehensive evaluation of cryoinjury and post-thaw functional capacity, key cellular integrity and physiological parameters were assessed using flow cytometry (Table 3). These assays move beyond conventional motility metrics by providing essential insight into fundamental functional components, including membrane stability, mitochondrial energy status, and susceptibility to oxidative damage. These metrics are crucial for detecting subtle, sub-lethal changes that might compromise subsequent fertility, even in the absence of marked motility decline.

#### 4.3.1. Viability and Acrosome Integrity

The percentages of viable spermatozoa (membrane-intact) did not differ significantly across the cooling rate groups (*p* = 0.08). Although numerically higher values were observed for the slow-cooling group (65.59% ± 2.15%) compared to the fast-cooling group (61.67% ± 2.01%), this difference failed to reach statistical significance. Similarly, the proportion of spermatozoa exhibiting disrupted acrosomes and intact acrosomes showed no significant variation between the cooling treatments (*p* > 0.05 for both).

#### 4.3.2. Mitochondrial and Oxidative Features

Mitochondrial membrane potential (MMP), assessed via the polarization ratio (polarized/depolarized), serves as a fundamental marker of mitochondrial integrity and the cell’s capacity for ATP production, which is essential for post-thaw motility. Cryopreservation is known to negatively impact these bioenergetic processes. Analysis of the post-thaw MMP polarization ratio revealed that the functional status of the mitochondria was maintained at statistically comparable levels across all three pre-freeze cooling rate groups (*p* > 0.05). Assessment of the intracellular oxidative status indicated no significant differences in the proportion of viable spermatozoa that were ROS- (i.e., did not express ROS) or were ROS+ (i.e., expressed ROS), among the cooling groups (*p* > 0.05 for both).

### 4.4. Summary of the Findings

In summary, the implementation of the fast-cooling protocol resulted in a statistically significant improvement in post-thaw total motility compared to the Slow protocol. This differential functional outcome was observed alongside a comprehensive evaluation of cellular integrity: plasma membrane integrity, acrosome status, mitochondrial polarization ratio, and ROS expression. These fundamental cellular parameters were maintained at statistically similar levels across all three distinct cooling rates evaluated, including the Fast protocol (25–30 °C/min). This lack of differential effect on key metrics of cellular integrity, contrasted with the improved motility, highlights a central and unexpected finding regarding the cryosurvival capacity of stallion spermatozoa under distinct controlled cooling kinetics.

## 5. Discussion

The primary objective of this study was to evaluate the impact of varied initial cooling rates- Slow (0.3 °C/min), Moderate (1 °C/min), and Fast (approximately 25–30 °C/min)- on the post-thaw quality of stallion semen using a novel, precision cooling technology. The most remarkable finding, which fundamentally challenges conventional cryobiological recommendations for equine semen, was the observation that the Fast cooling rate yielded statistically higher total motility post-thaw compared to the traditionally favored slow rate. This discussion aims to interpret this surprising outcome by examining the subtle differences in sperm function and the critical role of the novel precision cooling technology.

### 5.1. Resolution of the Cryobiological Paradox: Cold Shock Mitigation by Thermal Precision

Traditional cryopreservation protocols emphasize the necessity of slow cooling rates in the range of 20 °C to 5 °C to prevent cold shock damage, a phenomenon to which stallion spermatozoa are particularly susceptible [9,23]. The conventional expectation was that the Fast-cooling rate, exceeding 25 °C/min, would induce massive cold shock injury and result in significantly inferior quality. Cold shock is primarily attributed to thermotropic phase transitions in the plasma membrane lipids, where the membrane shifts from a liquid-crystalline to a gel phase, leading to the aggregation of intramembranous proteins and consequential leakage of intracellular contents [12]. Due to the heterogeneous lipid composition of the sperm membrane, this transition is a complex process occurring over a broad temperature range, resulting in domains of lipid phase separation and protein aggregation. It is during this unstable reorganization phase, specifically, that membrane permeability significantly increases and chilling-induced injury accumulates in a time-dependent manner [34,35]. The novel outcome suggests that the precision cooling technology used for pre-freeze chilling neutralized the expected detrimental effects of rapid cooling. This device, which establishes intimate contact between the cooling block and the entirety of the straw via conduction, ensures a highly uniform and linear thermal gradient, unlike conventional methods utilizing uncontrolled vapor or simple refrigeration. This methodological precision lends support to the hypothesis that the success of the Fast rate is due to thermal skipping of the membrane’s unstable phase transitions. By enabling rapid and uniform cooling, the technique is fast enough to traverse the critical state of lipid reorganization and phase separation, thereby preventing the accumulation of time-dependent chilling injury (such as irreversible intramembranous particle aggregation and consequential ion leakage) that typically accrues when the cell lingers in this vulnerable phase [12,34,35].

### 5.2. Functional Implications and Interpretation of Motility Variation

The observation that the Fast-cooling protocol (approximately 25–30 °C/min) resulted in significantly higher total motility post-thaw, set against the fact that no statistical variation was found for the clinically critical parameter, progressive motility (*p* = 0.320), requires careful interpretation. Progressive motility is broadly recognized as the superior predictor of clinical fertility in frozen-thawed stallion semen, with ≥30% pro-gressive motility often representing the minimal standard considered commercially acceptable [3,6,36]. The overall average post-thaw progressive motility across all cooling groups (26.9% to 30.99%) places the cohort precisely at this critical threshold. This finding is particularly important because the small sample size (*N* = 5) captured the inherent heterogeneity—encompassing both cryo-resistant (‘Good Freezers’) and cryo-sensitive (‘Poor Freezers’) ejaculates—characteristic of the equine population, as seen in Figure 2 [5,7]. Critically, since the clinically relevant parameter of progressive motility and key intracellular parameters showed no statistical difference, it is plausible that all protocols yielded equivalent fertility potential. Consequently, the key implication of the findings shifts toward operational efficiency. It is therefore hypothesized that the significant difference in total motility arises from distinct mechanisms of sub-lethal damage induced by the cooling rate kinetics.

*A.*  Proposed mechanism: Time-dependent injury and the performance of slow cooling

The lowest TM observed in the Slow protocol (0.3 °C/min, 45.01%) is best hypothesized as a consequence of cumulative injury caused by the extended processing time. This aligns with the cryobiological concept of “solution effects,” wherein cells suffer due to high solute concentrations in the unfrozen medium during slow cooling [8,16]. Specifically, Slow cooling inherently involves extended exposure to the Cryoprotective Agent (CPA, Glycerol). Prolonged exposure to Glycerol is known to potentially cause chemical toxicity, damaging the plasma membrane and cytoskeleton, independent of osmotic effects [22]. The extended duration required by the Slow protocol likely worsened these solution effects, resulting in a higher rate of lethal injury and the lowest overall TM. Conversely, the Fast protocol avoids this accumulation of time-dependent injury by rapidly traversing the initial cooling phase, preventing long-lasting exposure in the critical temperature range (19 °C to 8 °C) [13] where lipid phase transitions often lead to chilling injury in equine spermatozoa [12,37].

*B.*  Interpreting the viability trend and functional damage

While the overall integrity markers (acrosomal integrity, ROS, mitochondrial potential) were equivalent, the subtle trends in viability and circle motility suggest a shift in the nature of sublethal injury between the protocols. The Slow group demonstrated the numerically highest viability (65.59%), while the Fast group showed numerically the lowest (61.67%). Given the marginal statistical result (*p* = 0.08) and the small sample size (*N* = 5), this numerical pattern suggests a trend toward better membrane integrity maintenance in the Slow group. This supports the hypothesis that the prolonged stress of the Slow protocol inflicted specific kinetic damage on the flagellum or motility apparatus, rather than immediately lysing the cell. Cells thus remain technically “live” (membrane intact) but are functionally immotile or non-progressively motile (“live but immotile”). In contrast, the Fast protocol, by quickly bypassing the prolonged solution effects, successfully preserved more cells with minimal lethal damage, resulting in higher TM. However, these preserved cells may have sustained sufficient sub-lethal damage to limit them to non-progressive movement. This is reflected by the trend toward higher circle motility in the Fast group (*p* = 0.055). This higher TM, without corresponding improvement in progressive motility or core metabolic markers, is therefore hypothesized to reflect the successful maintenance of non-progressive viability in a population of cells that would have died completely under the extended stress of the Slow protocol.

### 5.3. Limitations and Future Perspectives

A major limitation of this study is the constrained sample size (*N* = 5 stallions), which limits the generalizability of the findings and increases the probability of a Type II error for subtle differences. Furthermore, the true success of any cryopreservation protocol must be validated by evaluating downstream effects, which were beyond the scope of this study:

DNA Integrity and Epigenetics: Cryopreservation is known to increase DNA fragmentation and alter DNA methylation patterns, potentially impacting subsequent embryonic development [17,18]. Since the different cooling rates resulted in distinct survival profiles (Fast favoring motility, Slow potentially favoring structural integrity), future studies must investigate whether the Fast protocol minimizes sublethal genetic or epigenetic damage. Similarly, while functional membrane integrity and ROS levels were maintained, further molecular profiling of the enzymatic antioxidant defense system and capacitation markers is warranted to fully elucidate the cellular mechanisms conferring this cryotolerance.

Delayed Cell Death (CIDOCD): Immediate post-thaw viability measurements may overestimate true survival, as delayed apoptosis and necrosis (CIDOCD) can occur up to 48 h post-thaw [38]. Longitudinal studies are needed to determine if the viability advantage of the Slow protocol, while marginal, translates to greater post-thaw stability.

Clinical Trials: Ultimately, validation of reproductive outcomes requires field fertility trials (artificial insemination). Given the equivalence in progressive motility, the clinical recommendation relies on the assumption that progressive motility is a sufficient predictive metric.

Clinical Application and Future Directions: Towards a “One-Stop” Cryopreservation Protocol: A significant practical implication of this study stems from the observation that the rapid, conduction-based cooling from room temperature to 5 °C did not compromise sperm quality. The cooling block used in this experiment is functionally analogous to the first-stage cooling block of the MTG device [31]. This finding opens a compelling direction for process optimization: it suggests the potential feasibility of a streamlined, “one-stop” cryopreservation protocol. Such a protocol would allow for the entire process, from initial cooling at room temperature through to the final freezing stages, to be conducted within a single device- eliminating the need for separate pre-cooling equipment. This would represent a substantial improvement in workflow efficiency for laboratories. Unpublished data from ongoing trials validate that direct placement into the MTG device’s initial block yields post-thaw sperm quality comparable to the fastest cooling group of this study, strongly supporting a simplified and accelerated clinical protocol.

## 6. Conclusions

The results demonstrate that the precision cooling technology effectively mitigates the cold shock traditionally associated with Fast pre-freeze cooling in stallion semen. Critically, since the quality of the clinically relevant parameter (progressive motility) remains statistically equivalent across the Moderate and Fast protocols, the optimal choice shifts decisively to operational efficiency. The ability to achieve clinically acceptable post-thaw sperm quality with the Fast or Moderate cooling rate, drastically reducing the required processing time (3–4 times faster), establishes a robust justification for adopting these optimized, time-saving protocols in commercial cryopreservation centers.

## Figures and Tables

**Table 1:** Summary of the initial characteristics of extended stallion semen samples (*N* = 15) prior to the initiation of the experimental cooling protocols.

Parameter	*N*	Mean	SEM	Median	Min	Max
Concentration (×10^6^/mL)	15	151.27	11.01	171.02	59.04	213.41
Total motility (%)	15	87.63	1.72	89.55	70.40	94.84
Progressive motility (%)	15	77.82	3.06	83.40	54.07	94.02
VCL (µm/s)	15	142.36	8.04	145.26	96.34	203.44

Abbreviations: VCL = Curvilinear velocity.

**Table 2:** Summary of sperm motility and kinematic parameters following processing with three distinct pre-freeze cooling rates. The table presents data assessed by CASA at two time-points: immediately after cooling (Post Cooling) and after thawing (Post Thaw). The results of the Aligned Rank Transform (ART) ANOVA are shown for the main effects of the processing stage, the cooling rate, and their interaction.

Parameter	Phase	Fast Cooling (Mean ± SEM)	Moderate Cooling (Mean ± SEM)	Slow Cooling (Mean ± SEM)	Effect	*F*-Value	*p*-Value
TM (%)	Post Cooling	84.49 ± 1.77	83.62 ± 2.43	82.73 ± 2.58	Phase	1008.55	<0.001
					Cooling Rate	5.86	0.004
	Post Thaw	51.87 ± 3.65	50.94 ± 3.27	45.01 ± 4.15	Phase: Cooling Rate	3.82	0.027
PM (%)	Post Cooling	71.28 ± 3.37	70.1 ± 4.15	69.84 ± 4.04	Phase	1094.66	<0.001
					Cooling Rate	1.64	0.201
	Post Thaw	30.99 ± 3.3	29.47 ± 3.75	26.9 ± 3.91	Phase: Cooling Rate	1.16	0.320
FM (%)	Post Cooling	31.34 ± 3.38	32.42 ± 3.64	32.77 ± 3.62	Phase	1167.45	<0.001
					Cooling Rate	0.18	0.834
	Post Thaw	7.98 ± 1.31	7.93 ± 1.25	7.47 ± 1.5	Phase: Cooling Rate	0.90	0.411
CM (%)	Post Cooling	7.51 ± 0.87	7.3 ± 0.88	6.67 ± 0.87	Phase	948.67	<0.001
					Cooling Rate	3.02	0.055
	Post Thaw	1.67 ± 0.42	1.24 ± 0.18	1.18 ± 0.23	Phase: Cooling Rate	1.53	0.224
VCL (µm/s)	Post Cooling	125.98 ± 6.33	125.32 ± 6.61	123.81 ± 6.87	Phase	1051.83	<0.001
					Cooling Rate	0.20	0.820
	Post Thaw	68.23 ± 4.53	66.89 ± 3.12	67.47 ± 3.99	Phase: Cooling Rate	0.09	0.911

Abbreviations: TM = Total motility, PM = Progressive motility, FM = Fast motility, CM = Circle motility, VCL = Curvilinear velocity.

**Table 3:** Summary of post-thaw sperm integrity and physiological parameters assessed by flow cytometry following processing with three distinct pre-freeze cooling rates.

Parameter	Fast Cooling (Mean ± SEM)	Moderate Cooling (Mean ± SEM)	Slow Cooling (Mean ± SEM)	*F*-Value	*p*-Value
Viability (%)	61.67 ± 2.01	64.68 ± 2.02	65.59 ± 2.15	2.76	0.08
Acrosome Disrupted (%)	1.16 ± 0.19	1.22 ± 0.25	1.17 ± 0.14	0.76	0.48
Acrosome Intact (%)	26.34 ± 2.01	26.15 ± 1.98	24.85 ± 1.97	0.32	0.73
MMP	0.96 ± 0.19	0.91 ± 0.16	1.18 ± 0.28	0.83	0.45
Viable ROS− (%)	16.5 ± 2.06	17.71 ± 1.96	16.09 ± 1.65	0.34	0.71
Viable ROS+ (%)	20.5 ± 2.47	18.53 ± 1.94	18.83 ± 1.77	0.68	0.52

Abbreviations: MMP = Mitochondrial membrane potential.

## Data Availability

None of the data were deposited in an official repository. Data are available upon request.
